# Chemotherapy, host response and molecular dynamics in periampullary cancer: the CHAMP study

**DOI:** 10.1186/s12885-020-06807-3

**Published:** 2020-04-15

**Authors:** Sofie Olsson Hau, Alexandra Petersson, Björn Nodin, Emelie Karnevi, Karolina Boman, Caroline Williamsson, Jakob Eberhard, Karin Leandersson, David Gisselsson, Margareta Heby, Karin Jirström

**Affiliations:** 1 Division of Oncology and Therapeutic Pathology, Department of Clinical Sciences, Lund University, SE-221 85 Lund, Sweden; 2 Division of Surgery, Department of Clinical Sciences, Lund University, Lund, Sweden; 3 Cancer Immunology, Department of Translational Medicine, Lund University, Malmö, Sweden; 4 Division of Clinical Genetics, Department of Laboratory Medicine, Lund University, Lund, Sweden

**Keywords:** Periampullary cancer, Pancreatic cancer, Chemotherapy, ctDNA, Immune response

## Abstract

**Background:**

Pancreatic cancer is a devastating disease with a dismal prognosis. Despite profound medical advances in systemic therapies for other types of aggressive tumours during recent years, a diagnosis of pancreatic cancer is still often synonymous with a fatal outcome. The term periampullary cancer includes pancreatic cancer and applies to the group of tumours found in proximity to the ampulla of Vater. Molecular events and immune response in the host during chemotherapy remain largely unexplored in this group of tumours. Therefore, the “Chemotherapy, Host Response and Molecular Dynamics in Periampullary Cancer (CHAMP)” study aims to monitor these processes to gain new insight into this perplexing disease.

**Methods:**

The CHAMP study is a prospective, single-arm observational study. All patients diagnosed with pancreatic or other periampullary adenocarcinoma undergoing adjuvant or palliative chemotherapy treatment in the Department of Oncology, Skåne University Hospital, are invited to participate. Clinical and pathological data will be compiled at study entry. A single tissue microarray (TMA) block is constructed for each patient with a resected tumour and blood samples are drawn before, during and after chemotherapy in order to sample peripheral blood mononuclear cells (PBMC), cytokines and circulating tumour DNA (ctDNA). Next generation sequencing will be performed on tumour tissue and ctDNA to detect changes in the clonal landscape over space and time.

**Discussion:**

Despite the recent emergence of some promising biomarkers for periampullary cancer, there has been a lack of success in clinical implementation. Cancer cells continuously adapt and become resistant to treatment during chemotherapy. To be able to keep pace with and hopefully overtake this rapid evolution we must, with the help of new diagnostic tools, be ready to adapt and alter treatment accordingly. It seems to us that the only way forward is to gain a better understanding of the dynamics of the disease during treatment. With insights gained from the CHAMP study we hope to find answers to key questions in this largely unexplored territory.

**Trial registration:**

This study has been registered 30th October 2018 at clinicaltrials.gov as NCT03724994.

## Background

### Pancreatic and periampullary cancer

Pancreatic cancer is one of the few malignancies where incidence approximates prevalence as the disease is almost uniformly fatal, often within 1 year. It is the most common tumour in the clinical entity called periampullary tumours, including tumours originating in the distal bile duct, pancreatic head, ampulla of Vater and the periampullary duodenum. Only 15–20% of the tumours are resectable at presentation, and for the remaining 80–85% of patients the situation is palliative. Adjuvant chemotherapy is the present standard of care, following resection, for pancreatic adenocarcinoma. In the palliative setting, median survival is approximately five to seven months with single-agent gemcitabine, or 8.5–11 months with more intense combination regimens such as gemcitabine/nanoparticle albumin-bound (nab)-paclitaxel and fluorouracil (5-FU)/irinotecan/oxaliplatin (FOLFIRINOX) [1, 2]. However, apart from the prognostic information provided by standard histopathological parameters, no predictive biomarkers for response to neoadjuvant, adjuvant or palliative chemotherapy have yet been introduced into clinical practice. Moreover, although the vast majority of pancreatic cancers contain somatic mutations, there is still a complete lack of “actionable” molecular targets, also immunotherapy has not as yet yielded any substantial clinical benefit for patients with pancreatic cancer [3, 4].

### Tumour heterogeneity

Despite an increasing battery of successful systemic therapies available for the treatment of irresectable or disseminated cancers, tumour response is often transient. Tumours, like living organisms, evolve and continuously adapt to overcome treatment by Darwinian selection of pre-existing resistant clones and/or through transcriptional reprogramming [5–8]. Although these dynamics and the evolutionary nature of tumours is well-recognized, systemic therapies are typically administered in a static, linear fashion, through protocols that a priori fix the drug(s), dosage and timing. Adding to this, biomarker analyses, if applicable, are only performed on one selected sample from a pre-treatment surgical specimen or, in irresectable cases, a biopsy. In a similar fashion, the majority of biomarker studies only consider clinical outcome in relation to analysis of one sample. This is likely the reason why, despite the plethora of scientific literature on promising biomarker candidates, very few make it into clinical protocols and add value to the treatment of cancer patients.

Cancer evolution is reflected in both spatial and temporal tumour heterogeneity. Spatial tumour heterogeneity refers to differences within a single tumour mass at a given moment and temporal heterogeneity refers to changes over time [9, 10]. Plasma-derived circulating tumour DNA (ctDNA), shed from primary tumours and/or their metastases, provides a good readout for identifying emerging treatment-resistant clones, for tracking the development of the intratumoural heterogeneity of the cancer, and for predicting responses and resistance to treatment [11, 12] . The quantity of ctDNA is also associated with patient survival [13]. Immune responses not only correlate with molecular characteristics of pancreatic cancer [14, 15], but are also integral for anti-cancer adaptation, for preventing as well as hindering the development of life-threatening malignant cells. Paradoxical interactions may also occur between the immune system and cancer cells, where the immune system, rather than playing a suppressing role in cancer development instead helps promote tumour growth both locally and systemically [12]. How this interplay is affected by chemotherapy is still relatively unknown.

In summary, despite great advances in oncology research, the molecular events within tumours and the relationship between cancer cells and the immune system occurring during systemic therapy remain strikingly unexplored. Therefore, understanding the clinical consequences of spatial and temporal tumour heterogeneity is essential for designing and deciding upon effective therapeutic strategies and more precise diagnostics. While numerous studies exist on the evolutionary principles underlying malignant tumours [16, 17], very few of these have been performed in a clinical, prospective setting, using comprehensive registries and biobanks.

## Objectives

We hypothesize that there is a functional interplay between the clonal evolution of cancer cells and the local and systemic immune response against pancreatic cancer. Furthermore, we postulate that the outcome of this interaction is affected by treatment and may even be a crucial determinant of treatment response. The CHAMP-study is an initiative to decipher the nature and extent of this cancer-host interplay, in tumour tissue and in blood, using a longitudinal perspective. We hypothesize that the therapeutic resilience of pancreatic cancer can be overcome by dynamic monitoring of the disease, combined with the use of tailored combinations of immunotherapy and chemotherapy, and a readiness to alter treatment depending on the clonal dynamics of the tumour as well as the host response.

The specific objectives are:

- To examine the associations between the spatial heterogeneity of cancer cell genotypes and phenotypes with the inflammatory tumour microenvironment and stromal characteristics in resected tumours
- To examine the associations between spatial genetic heterogeneity and temporal genetic heterogeneity in cases with resected disease
- To examine the prognostic value of systemic circulating immune cells
- To examine the associations between characteristics and heterogeneity of the inflammatory microenvironment with host response during treatment
- To identify patterns of dynamic temporal variations and correlations between genetic alterations and host immune response that impact patient survival and disease progression
- To examine the associations of circulating concentrations of ctDNA with the spatial and temporal heterogeneity of genetic alterations and the host immune response.
- To examine the prognostic value of circulating levels of ctDNA

## Methods/design

### Study design/eligibility criteria

The CHAMP-study is a prospective, single-arm observational study. All patients with a histologically or cytologically confirmed diagnosis of pancreatic or other periampullary adenocarcinoma undergoing adjuvant or palliative chemotherapy treatment in the Department of Oncology, Skåne University Hospital are invited to participate. A total enrolment of 150 patients is planned. This study has been registered in clinicaltrials.gov as NCT03724994.

The main exclusion criteria are: 1. patients having another concomitant life-threatening disease and 2. patients who are unable to receive chemotherapy.

### Measurements

**Fig. 1:**
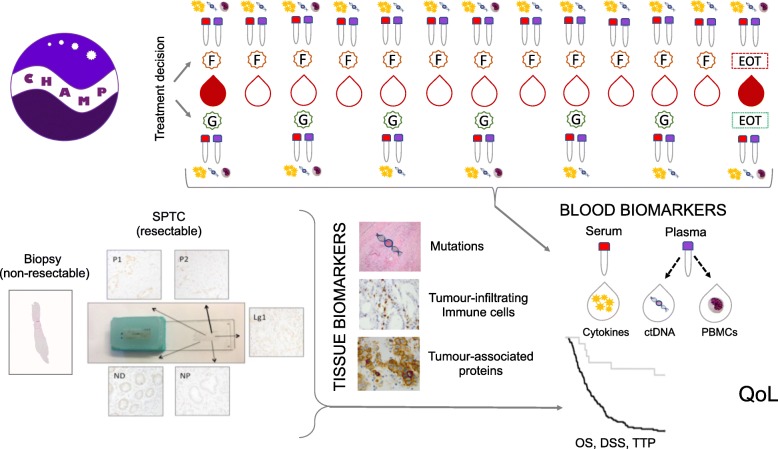
Flowchart describing the study inclusion procedure. F and G are abbreviations of FOLFIRINOX and gemcitabine. These are examples of regimens, others such as nab-paclitaxel follow similar schedules. DSS = disease specific survival, EOT = end of treatment, OS = overall survival, QoL = Quality of life, TTP = time to progression, SPTC = single patient tissue chip, ctDNA = circulating tumour DNA, PBMCs = Peripheral blood mononuclear cells

A flowchart of the study is shown in Fig. 1 and a timeline of all planned procedures for each included participant is shown in Tables 1 and 2. Clinical and pathology data are compiled at study entry. Radiological and clinical follow ups will be performed every 3 months. Primary endpoint is overall survival, secondary endpoints will be disease specific survival, time to progression, and quality of life (EORTC-QLQ-PAN26). Serial sampling of blood during chemotherapy treatment is performed by a dedicated research nurse along with the clinical routine sampling. Plasma and serum samples for analysis of ctDNA and cytokines, respectively, are drawn before the start of chemotherapy, and prior to each additional course of chemotherapy (monthly e.g. gemcitabine based regimens or biweekly e.g. combination regimen FOLFIRINOX), and after the last course of treatment. Peripheral blood mononuclear cells (PBMC) are isolated from buffy coat in plasma vials before start of chemotherapy, before the second or third (monthly or biweekly, respectively) course of chemotherapy, before the fourth or seventh course of chemotherapy, and after the last course of treatment.

**Table 1:**
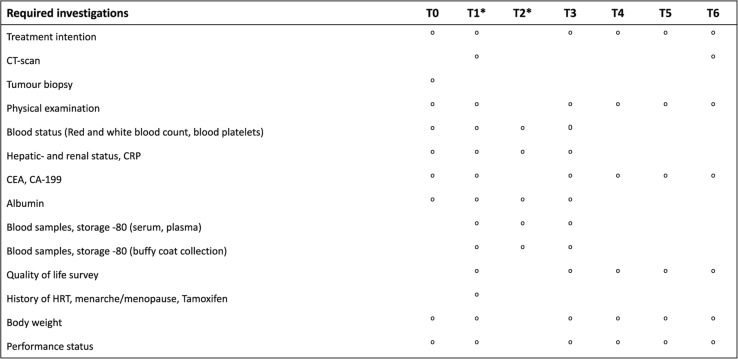
Procedure timeline in the adjuvant setting.

**Table 2:**
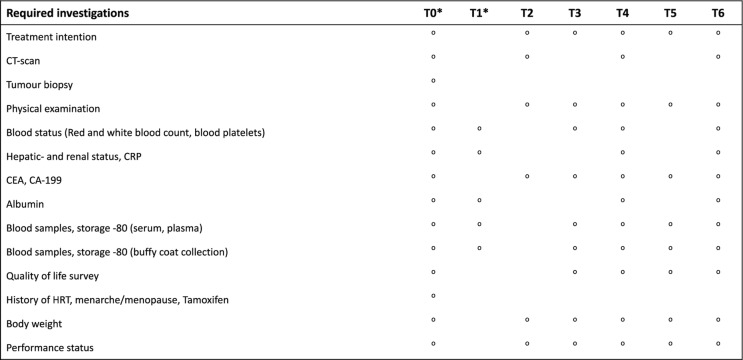
Procedure timeline in the palliative setting.

## Methods

### Single patient tissue Chip

**Fig. 2:**
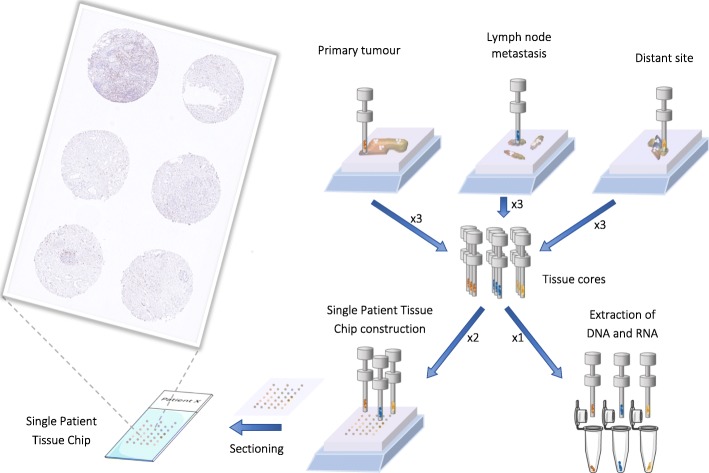
Methodological overview of the construction of a “Single patient tissue chip” from a resected tumour. This figure was created using Servier Medical Art templates, which are licensed under a Creative Commons Attribution 3.0 Unported License; https://creativecommons.org/licenses/by/3.0/

To account for spatial tumour heterogeneity in resected tumours, we have developed a concept called “Single Patient Tissue Chip” (SPTC); a single tissue microarray (TMA) block that is constructed for each patient with a resected tumour in the CHAMP-study (Fig. 2). The SPTC contains several 1 mm tissue cores from all archival paraffin blocks with a sufficient amount of primary tumour, stroma, normal tissue, and 1–2 cores from each present lymph node metastasis of sufficient size. Alongside with the construction of SPTCs, additional tissue cores are taken from each of these areas for DNA and RNA extraction and next generation sequencing.

### Immunohistochemical analyses

Quantitative and compartment-specific analyses of immune cells will be performed by immunohistochemical analysis on biopsies (depending on tissue quantity and quality) and on SPTCs. Multiplexed immunofluorescence imaging [18], using a customized panel of several immune cell markers, will be applied when feasible. Mismatch repair (MMR) status will be evaluated by immunohistochemical analysis of MMR proteins MLH1, PMS2, MSH2 and MSH6.

### Mutation analyses

The AllPrep DNA/RNA FFPE kit (Qiagen, Hilden, Germany) and the QIAsymphony DSP Circulating DNA Kit (Qiagen) will be used for extraction and purification of DNA and RNA from tumour tissue and DNA from plasma, respectively. Targeted deep sequencing (www.illumina.com) using a comprehensive oncoprofiling approach with > 500 cancer related genes will be applied for the mutation analyses of tumour tissue. For ctDNA, an appropriate panel will be selected when the prevalence of mutations in resected specimens and biopsies have been further delineated. The mutational load will be calculated in both tissue and plasma samples.

### Cytokines, PBMCs

Freezing of PBMCs will be performed according to standard procedures. Changes in immune cell subsets among PBMCs will be performed by multiparameter flow cytometry (FACSVerse), with a panel of markers including a) T-cell subsets b) B cells c) natural killer cell subsets, defined by expression patterns of activating and inhibitory receptors, and d) myeloid cell subsets. Immunological analyses of peripheral blood cells will additionally be performed with Mass Cytometry analysis, using the single-cell proteomics platform CyTOF™ (Fluidigm.Inc) [19], on three blood samples from each patient (pre-, on- and post-chemotherapy). Serum samples will be used to measure levels of a panel of cytokines and chemokines, using Microarray cytokine assay kits such as Evidence Investigator and the Cytokine Array I and High sensitivity kit (Randox, Antrim, UK).

### Quality control and assurance

The study is conducted in accordance with the GCP guidelines for quality control and quality assurance. The sponsor reviews the CRF regularly. The web-based application REDCap will be used to manage all data generated, including an electronic case report form (eCRF).

### Data analysis

The impact of different mutations, immune cell subsets and combinations thereof, in resected tumours and biopsies on overall and disease-free survival and time to progression, will be analyzed by the Kaplan-Meier method and the log- rank test. Univariable and multivariable Cox regression models will be applied to calculate hazard ratios for progression, relapse and death. The more complex data analyses related to variations and intercorrelations of mutational load, cytokines and immune cell signatures, in tissue samples and pre-, on- and post-therapy blood samples from responders and non-responders, will be performed in-house. Targeted and whole-exome sequencing will be followed by clonal deconvolution and the construction of cancer cell phylogenetic trees according to Karlsson et al. [20] using a customized R-script. Individual somatic mutations and phylogenetic data will be correlated to ctDNA mutational profiles, clinical data and the panorama of infiltrating immune cells.

## Discussion

Although the majority of metastatic patients have a poor or short-lived response to chemotherapy, with an overall survival of 8 to 11 months with chemotherapy versus 3 to 6 without, some (18,6%) are deemed long-term survivors, living more than 18 months from diagnosis [2]. The factors contributing to long-term survival remain largely unknown.

In the time span between a cancer diagnosis and the point where treatment can be declared effective or not, lies a vast, unexplored land of molecular events and host responses. Through the CHAMP-study we aim to gain insight into this complex process. For pancreatic cancer patients there is a striking lack of success to implement clinically useful tools for complementary diagnostics. Thus, it seems that dynamic disease monitoring is the only way forward in order to help researchers and clinicians refine their theories and address some of the big unknowns.

Worldwide, there are several ongoing studies aimed at shedding light on pancreatic cancer by the mean of longitudinal observational studies. The clinical trials COMPASS (https://clinicaltrials.gov/ct2/show/record/NCT02750657) and BIOPAC (https://clinicaltrials.gov/ct2/show/record/NCT03311776), are two of the ongoing efforts alongside the CHAMP study trying to gain insight into the clonal dynamics of this perplexing disease. COMPASS looks for genomic characteristics to identify patient subgroups at time of diagnosis enabling refined selection of treatment. BIOPAC is a large study that utilizes on-treatment blood sampling to find new biomarkers to improve patient outcome and enable diagnosis at an earlier stage. An additional strength of CHAMP is the use of single patient tissue chips along with on-treatment blood sampling for examining spatial and temporal heterogeneity together with the interplay of both local and systemic immunological responses during treatment.

The design of the prospective study and the establishment of a biobank through recurrent blood sampling is well-suited for acquisition of real data in real time regarding the functional interplay between the shifting clonal dynamics of cancer and the immune system during treatment. Thus, we anticipate that the acquired knowledge will be instrumental for future development of not only diagnostic tools but also adaptive treatment strategies, including immune activating drugs, that may be able to keep pace with, or possibly even outrun, the rapid evolution of resistant cancer cells. The design of the CHAMP-study is also well suited for other types of aggressive cancer where effective treatment and diagnostic tools are few or lacking. Our hope is that this type of dynamic, prospective, on-treatment biomarker focused design can be applied in other cancer centers both nationally and internationally, thus spurring and speeding up collaborative research efforts so that we will finally be able to make headway on the path towards understanding and improving outcome for patients with this fatal disease.

### Trial status

Initiated in October 2018.

## Data Availability

Not applicable.

## Abbreviations

CHAMP: Chemotherapy, host response and molecular dynamics in periampullary cancer
CRF: Case report form
ctDNA: Circulating tumour DNA
FOLFIRINOX: Fluorouracil (5-FU)/irinotecan/oxaliplatin
GCP: Good clinical practise
MMR: Mismatch repair
PBMC: Peripheral blood mononuclear cells
SPTC: Single patient tissue chip
TMA: Tissue micro array
